# Trauma to the Eye: Diffusion Restriction on MRI as a Surrogate Marker for Blindness

**DOI:** 10.3390/tomography9010033

**Published:** 2023-02-16

**Authors:** Andreas Stahl, Norbert Hosten

**Affiliations:** 1 Department of Ophthalmology, University Medicine Greifswald, 17475 Greifswald, Germany; 2 Department of Radiology, University Medicine Greifswald, 17475 Greifswald, Germany

**Keywords:** traumatic brain injury, sight loss, optic nerve, MRI, diffusion-weighted imaging

## Abstract

Traumatic optic nerve injury may lead to almost instantaneous blindness. We describe a case of sight loss after a perforating injury to the eye. The case is unusual in that the patient remained conscious and the trauma to the eye was isolated. A full ophthalmological examination was therefore possible within hours as well as early magnetic resonance imaging of the facial skull. High-quality T1-weighted, T2-weighted, and diffusion-weighted imaging could be acquired. The latter included apparent diffusion coefficient maps. There was a loss of the subarachnoid space of the optic nerve, fluid in the retrobulbar fat of the affected eye, and signal changes in the optic nerve. Previous work has been contradictory on the signal of the optic nerve on apparent diffusion coefficient maps in sight loss, with an increase seen by one group and a decrease seen by another. Signal loss on the apparent diffusion coefficient map was seen in the case described here. Signal loss on apparent diffusion coefficient maps may thus be used as a surrogate marker of sight loss in patients who are unconscious or otherwise unable to cooperate in ophthalmological exams.

## 1. Introduction

Traumatic brain injuries with or without a fracture of the optic canal can lead to damage of the optic nerve, which manifests itself in impaired vision and even blindness. Direct impact is not a prerequisite for blindness. The cause of damage was often difficult to determine before MR imaging became available: individual patients who survived went blind; however, no histological clarification was possible in these patients. Other patients died, and here histological clarification could be obtained; whether it caused or would have caused blindness, however, remained unclear [1]. Isolated cases described in the older literature, in which primarily patients who had initially survived died later in the course of the trauma from contusion pneumonia, have provided some correlation between ophthalmological and histological findings. With today’s improved imaging, detection of a fractured optic canal is much improved by CT scans in comparison to X-ray images; MRI and especially diffusion-weighted imaging can detect trauma-induced changes of the optic nerve early in the progression of disease. Newer research has correlated these findings to those seen in trauma to the brain. The exact appearance of optic nerve changes seen in blindness, however, is controversial.

In the case described here, both a full ophthalmological examination and CT/MR imaging including diffusion-weighted imaging were possible in a young patient with one-sided vision loss after local trauma to the right eye. The findings underline the importance of evaluating the orbits in patients with trauma to the head, who have undergone MR imaging of the brain. Sedation, coma, raised intracranial pressure, or mind-altering drugs may prevent full ophthalmological examination in these patients, where MRI then may indicate vision loss.

## 2. Case Report

**Figure 1:**
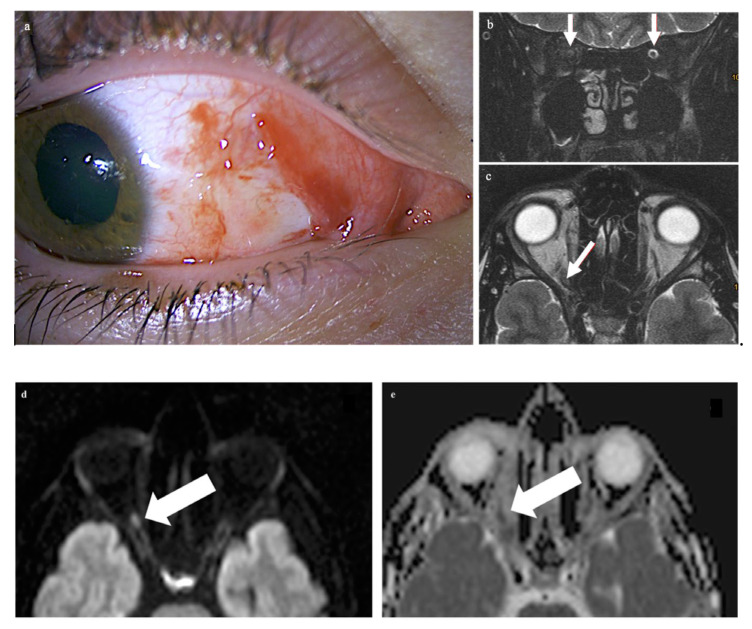

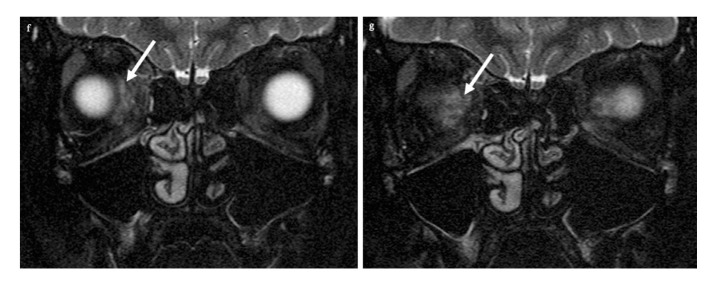
Examination of the right eye revealed a superficial conjunctival wound nasally and a dilated pupil (**a**). On the MRI scan, there is loss of the subarachnoid space in the right optic nerve (arrows in panel (**b**): the subarachnoid space of the left optic nerve is normal). The respective transverse T2-weighted image shows a central signal increase in the optic nerve (arrow in (**c**)). On the diffusion-weighted imaging, a punctuate signal increase in the optic nerve in the same location is seen (arrow in (**d**)); the corresponding ADC map shows signal loss in the same location (arrow in (**e**)). The globe as well as the left eye and orbit are unchanged, while there is a diffuse signal increase (arrows) in the right retrobulbar space nasally (**f**,**g**).

A 21-year-old male patient presented with vision loss in his right eye after trauma. He had injured himself with a metal gardening device and suffered parabulbar penetration of a metal rod into the orbit with only minimal ocular trauma. The metal rod was thin, but with a blunt end; it directly hit and tore into the orbit. Examination of the right eye revealed a superficial conjunctival wound nasally and a dilated pupil (Figure 1a). Ophthalmological examination of the left eye was normal. The globe was not injured. The cornea, lens, retina, and optic nerve head appeared normal upon ophthalmologic examination. Functional ocular examination, however, showed no light perception and a relative afferent pupillary defect. 

CT of the facial skull showed neither fractures nor foreign bodies, metallic or otherwise (Somatom Excite, Siemens Healthineers, Erlangen, Germany). The optic canal was normal. MR imaging was performed 4 hours after the trauma on a 1.5 T imager (Symphony, Siemens Healthineers, Erlangen, Germany) with a standard head coil. Plain T1w spin-echo images in the transverse orientation (TR 500 ms, TE 8.8 ms) and plain T2w turbo spin-echo transverse and coronal images with and without fat saturation (TR 4.000 ms and TE 128 ms) by prepulse were obtained in a 2 mm slice thickness. For DWI and ADC maps, a transverse orientation was chosen (5 mm slice thickness, TR 2500, TE 80 ms, and b-value of 1.000 s/mm^2^). Imaging demonstrated on coronary T2-weighted fat-saturated MRI loss of the subarachnoid space in the right optic nerve (white arrows in panel b: the subarachnoid space of the left optic nerve is normal) while the respective transverse T2-weighted image revealed slight optic nerve distention with a central signal increase (white arrow in c) right frontally from the optic canal. Diffusion-weighted imaging showed a punctuate signal increase in the optic nerve in the same location (d); the corresponding ADC map had signal loss in the same location (e). The globe was unchanged, while the retrobulbar space showed a diffuse signal increase nasally (f,g). The left eye and orbit were normal.

Treatment was purely local. Sight loss was permanent. There was no follow-up.

## 3. Discussion

The macroscopical anatomy of the optic nerves has been textbook knowledge since the transition from the 19th to the 20th century. As part of the brain, the optic nerve is covered by the arachnoid and pia mater. The nerve comes into close contact with the dura mater upon entering the optic canal. There, the dura mater is the periosteum of the bone. Upon leaving the optic canal and entering the orbit, the dura mater splits into two leaves. The outer layer becomes the periosteum of the bones constituting the orbit while the inner layer covers the pia mater at its outside and accompanies the optic nerve complex until it arrives at the sclera. The influences of this anatomy on traumatic optic nerve injury have been better understood in the following decades from ophthalmological, neurosurgical, and anatomical research. With the development of non-invasive imaging, this knowledge was transferred to in vivo studies of trauma patients.

Regarding traumatic injury of the optic nerve, anatomic descriptions have long focused on the relationship between the optic canal, the optic nerve, the dural, pia, and arachnoid, and the ophthalmic arteries and the perforators originating from it and nourishing the nerve from the periphery to the center. From these, Seitz [2] justified the fact that the optic nerve in the rear part of the optic canal seems primarily affected by trauma, with the following anatomical peculiarities: upon entering the bony canal, the optic fascicle is surrounded by the pia, the arachnoid, and the dura. In the area of the roof of the canal, the dura is firmly attached to the bone, forming its periosteum. Fixation is strongest at the inner mouth of the canal. Otherwise, the dura is freer to move. In the area of the posterior roof of the canal, small vessels penetrate through the dural sheath into the optic nerve and supply it. In the case of blunt trauma to the head or to the face, the fixated part of the optic nerve moves less than the non-fixated part. Small penetrating vessels may thus suffer shear stress. In more focused penetrating trauma, the mechanism of injury may be direct. As reasons for traumatic blindness from blunt trauma the following have been discussed: (1) shearing and bisection of small perforating arteries may lead to infarction of the nerve and to bleeding, both into the subarachnoid space and into the nerve proper; (2) Post-traumatic swelling of the nerve could compress nutritive arteries and this can lead to infarction [2]; (3) bisection of the optic nerve itself; and (4) swelling may also be caused by optic canal fracture [1]. Seitz presents histologic sections through optic nerves from patients with traumatic blindness. Disintegration, loosening, and destruction of axons and myelination were seen [2]. Hughes treats lesions of the anterior optic nerve as a somewhat different entity and proposes torsion and stretching of the nerve caused by the forced motion of the globe [3]. A more recent comprehensive review, drawing on neurosurgical and other literature, was given by Steinsapir and Goldberg [4] in a paper on diagnostic and therapeutic aspects of traumatic optic nerve injury. For interpreting MR images, the following measurements may be helpful: the optic canal has a length of 5.5 to 16.0 mm. The S-shaped intraorbital nerve measures 25 mm and bridges a distance between the globe and orbital apex of 18 mm. The extra length of the optic nerve gives mobility and is in contrast to the nerve’s tight connection to the dura/periosteum inside the canal. This helps prevent avulsion. The distance between the proximal opening of the optic canal and the optic chiasm is quoted by Steinsapir and Goldberg as 3 to 16 mm. Anterior and posterior optic nerve injury, with the latter mostly but not exclusively being indirect, occurs anteriorly and posteriorly to where the central retinal artery enters the optic nerve. Steinsapir and Goldberg highlight the importance of this distinction by quoting anecdotal reports of successful surgical treatment of intra-sheath hemorrhage in posterior optic nerve injury.

While traumatic optic nerve injury has mainly been a topic of clinical and anatomical research, the refinement of imaging from X-ray images, computed tomography, and magnetic resonance imaging to diffusion-weighted magnetic resonance imaging has added possibilities of making small contributions to treatment decisions in cases where vision cannot be fully assessed clinically. 

Optic canal fracture has been difficult to diagnose on X-ray images and its percentual contribution to traumatic sight loss has been controversial [1]. Somewhat astonishingly, it met even less interest after the introduction of sectional imaging by CT, presumably because the depiction of fractures was so convincing without much explanation. Involvement of the orbit is frequent in trauma patients. From a group of 1061 patients with CT examinations in a level 1 trauma center, 274 patients had orbital fractures while 101 patients had only orbital soft tissue injuries [5]. Fracture of the optic canal was not specified in this paper. Lee et al. [6] found impingement of the optic nerve by optic canal fragments in 2.7% of 150 patients in his series of orbital trauma. Menjot de Champfleur et al. [7] in their paper advocating MRI as the imaging modality of choice for the orbits indicate, as additional causes, traumatic lesions of the optic nerve inside the canal and intraconal or subperiosteal hematoma. These changes may ultimately lead to compression of the optic nerve and the development of compartment syndrome. If a fracture in the tip of the orbit is detected in CT, MRI may be used to directly demonstrate damage to the optic nerve (Becker et al. [8]). In the CT and MRI case presented by these authors, axonal damage to the optic nerve was described as a result of a skull base fracture. This was hypointense in the CISS sequence, which was attributed to microscopic hemorrhage. The presented case showed a diffusion restriction with the widening of the nerve, compatible, according to the authors, with axonal damage. The patient had permanent unilateral blindness.

Diffusion-weighted imaging today is considered valuable in patients with ischemic stroke. Changes are early and easily detectable in the b1000 images and in ADC maps. Cytotoxic edema is characterized by a signal increase in the b1000 image and in low ADC values, causing hypointensity in the ADC maps. The phenomenon was described qualitatively, but with opposing results by Yang et al. [9] and by Bodanapally et al. [10]. The changes described by Bodanapally et al. were pronounced in the posterior part of the optic nerve. Similar phenomena had previously been described by others for patients with head trauma (Galloway et al. [11]). Infarction of the optic nerve, caused by shearing and tearing of perforating arteries or induced by compartment syndrome in the optic canal as described above may be the cause. Galloway et al. correlated ADC values in the peripheral white matter of the hemispheres with the outcome after traumatic brain injury. Lower ADC values (visualized as low signal “dark” areas on ADC maps) were found in a group with severe head trauma and poor outcomes. The authors assigned these ADC values, based on clinical follow-up, a certain prognostic value regarding recovery. Bodanapally et al. transferred this concept to imaging of the optic nerve [10]. They examined 29 patients with traumatic optic neuropathy. MRI with DWI was available in all of the patients, and ADC maps were available in 25 patients. They found that diffusion restriction expressed by a decrease in ADC was specific for traumatic optic neuropathy, but at 27.6%, it was not sensitive. Yang et al. [9], however, found significantly increased ADC values in the same clinical entity.

There is probably some uncertainty regarding these findings. Post-traumatic changes in the brain are frequent at the border between grey and white matter. Diffuse axonal injury, a type of shearing injury of the brain, is a sequela. In the brain, it is mainly caused by deceleration, leads to punctate hemorrhages in white matter, and is best seen on susceptibility-weighted imaging. Susceptibility imaging, however, is hardly feasible in the orbit due to susceptibility artifacts from bone and the neighboring air-filled spaces of the paranasal sinuses. The optic nerve is pure white matter, and shearing injury would be caused by different acceleration/deceleration of the optic nerve in the optic canal and outside of it, as discussed. Shearing of penetrating pial arteries in the optic canal, on the other hand, would lead to infarction, as would swelling of the nerve there. The limited number of reported series of ADC MR imaging in traumatic optic neuropathy, all of them retrospective with the clinical indication for MRI and obtained at different times after injury, may account for discrepancies in reported findings.

Hemorrhage into the optic nerve sheath was mentioned by Steinsapir and Goldberg [4,12], who also described anecdotic evidence, that hemorrhage into the subarachnoid space of the optic nerve can constitute an indication for surgery. The general use of corticosteroids was not recommended by these authors. While in the case presented here, 2 mm slices were acquired, FIESTA or CISS sequences in 1 mm thickness may prove advantageous in delineating the subarachnoid space. We used 2 mm. Another post-traumatic condition where surgery may be considered is intraconal hematoma of the orbit [13]. As vision was lost completely immediately after the trauma and no other condition like a hematoma or dislodged bone fragments were seen on imaging, surgery was not considered in this case.

**Figure 2:**
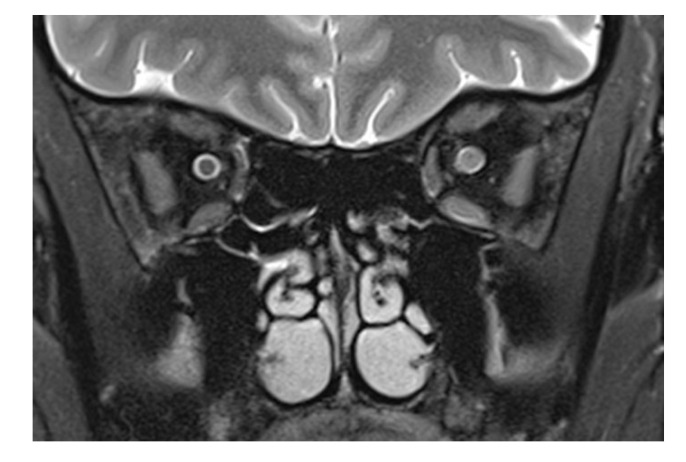
T2-weighted frontal image in a multiple sclerosis patient with optic neuritis. While the subarachnoid space has a normal diameter on the left, an enlarged optic nerve encroaches in the same space, recognizable by the smaller liquor-filled rim of the nerve. Leakage of cerebrospinal liquor after direct laceration of the sheath, however, cannot be counted out. The diffuse signal increase of the nasal retrobulbar space in Figure 1f,g may be explained by cerebrospinal liquor leaking from an injured optic nerve sheath and by edema.

The patient presented here had direct trauma to the right eye, and MRI was performed after full ophthalmological examination, only hours after the trauma in a conscious and cooperative patient. The trauma was a “perforating stab wound” [4] but missing the globe. The only sequela clinically visible was a conjunctival tear. CT excluded soft tissue changes, fracture, and foreign bodies in the globe and orbit. On MRI, changes in the optic nerve (and impossible to be seen on CT) were noted. This structure showed an opacification of the subarachnoid space, compatible with hemorrhage: an increase in the T2 signal of the optic nerve anterior to the inner opening of the optic canal; an increase in signal intensity on the diffusion-weighted b1000 image; and a decreased signal in the ADC map in the same location. The obliteration of the subarachnoid space around the optic nerve was different from what is commonly seen in the enlargement of an optic nerve. (For comparison, Figure 2 shows a T2-weighted image of an inflamed optic nerve in a patient with multiple sclerosis. There, the nerve supplants the subarachnoid space, which in comparison to the right and unaffected side is less wide).

While the case allows for a correlation of imaging findings and the clinical situation (signal increase in the b1000 image; signal loss on the DWI map; and complete and immediate sight loss), one can only speculate on the reason for the loss of sight. Some traction on the optic nerve seems provable in a penetrating stab wound from the periphery of the orbit. This would result in ischemic damage and should be concordant with the immediacy of the inflicted sight loss. In ischemic damage, small vessels traversing the subarachnoid space are torn and this could result in signal changes. As the diameter of the optic nerve seemed normal, a swollen nerve causing obliteration of the space seems less probable.

## 4. Conclusions

This case demonstrates severe optic nerve injury following parabulbar penetration of a metal rod into the orbit with only minimal ocular trauma and supports the role of orbital imaging when slit lamp ophthalmologic examination and functional ophthalmologic testing yield contradicting results. Radiological attention should be focused on the appearance of the optic nerve and the subarachnoid space inside its sheath on T2-W and on diffusion-weighted images and ADC maps. In patients who are unconscious or otherwise compromised regarding ophthalmological examination, a signal loss of the posterior optic nerve on ADC maps may serve as a surrogate for blindness. There is anecdotal evidence that patients with hemorrhage into the nerve’s subarachnoid space may benefit from surgical decompression.
